# PM_2.5_ induced liver lipid metabolic disorders in C57BL/6J mice

**DOI:** 10.3389/fendo.2023.1212291

**Published:** 2023-09-14

**Authors:** Chenxiao Zhang, Tengfei Ma, Chang Liu, Ding Ma, Jian Wang, Meng Liu, Jinjun Ran, Xueting Wang, Xiaobei Deng

**Affiliations:** ^1^ School of Public Health, Shanghai Jiao Tong University School of Medicine, Shanghai, China; ^2^ College of Basic Sciences, Shanghai Jiao Tong University School of Medicine, Shanghai, China; ^3^ Department of Cardiology, Renji Hospital, Shanghai Jiao Tong University School of Medicine, Shanghai, China; ^4^ Department of Cardiology, Tongren Hospital, Shanghai Jiao Tong University School of Medicine, Shanghai, China

**Keywords:** particulate matter, metabolomics, transcriptomics, hepatic steatosis, PPARα, PPARγ, SREBP1

## Abstract

PM_2.5_ can cause adverse health effects via several pathways, such as inducing pulmonary and systemic inflammation, penetration into circulation, and activation of the autonomic nervous system. In particular, the impact of PM_2.5_ exposure on the liver, which plays an important role in metabolism and detoxification to maintain internal environment homeostasis, is getting more attention in recent years. In the present study, C57BL/6J mice were randomly assigned and treated with PM_2.5_ suspension and PBS solution for 8 weeks. Then, hepatic tissue was prepared and identified by metabolomics analysis and transcriptomics analysis. PM_2.5_ exposure can cause extensive metabolic disturbances, particularly in lipid and amino acids metabolic dysregulation.128 differential expression metabolites (DEMs) and 502 differently expressed genes (DEGs) between the PM_2.5_ exposure group and control group were detected. The Kyoto Encyclopedia of Genes and Genomes (KEGG) enrichment analyses showed that DEGs were significantly enriched in two disease pathways, non-alcoholic fatty liver disease (NAFLD) and type II diabetes mellitus (T2DM), and three signaling pathways, which are TGF-beta signaling, AMPK signaling, and mTOR signaling. Besides, further detection of acylcarnitine levels revealed accumulation in liver tissue, which caused restricted lipid consumption. Furthermore, lipid droplet accumulation in the liver was confirmed by Oil Red O staining, suggesting hepatic steatosis. Moreover, the aberrant expression of three key transcription factors revealed the potential regulatory effects in lipid metabolic disorders, the peroxisomal proliferative agent-activated receptors (PPARs) including PPARα and PPARγ is inhibited, and the activated sterol regulator-binding protein 1 (SREBP1) is overexpressed. Our results provide a novel molecular and genetic basis for a better understanding of the mechanisms of PM_2.5_ exposure-induced hepatic metabolic diseases, especially in lipid metabolism.

## 1 Introduction

With the rapid development of the world population, economy, and industrialization, energy consumption also grows increasingly. Air pollution has become an urgent global health problem (1, 2). Particulate matter 2.5 (particulate matter with an aerodynamic diameter of ≤ 2.5 µm, PM_2.5_), as one of the main pollutants, can penetrate the respiratory barrier simply with its extremely tiny particle diameter by absorbing and combining with toxic compounds (3, 4). Then it can reach almost all organs through the bloodstream (5). Many epidemiological, animal, and *in vitro* studies found that health effects can be caused by exposure to airborne PM_2.5_, such as increased morbidity and mortality rates, even at concentrations meeting the environmental criteria (6–8). PM_2.5_ can lead to extensive inflammation (9) and irreversible damage in almost all systems (10, 11), which causes respiratory diseases like asthma and lung cancer (12, 13), circulatory diseases like ventricular hypertrophy and heart disease (14), and neurological diseases like retinopathy (15, 16).

As an important metabolic center of the body, the liver plays an important role in maintaining the homeostasis of the internal environment and energy system (17). It participates in the regulations of the synthesis, storage, decomposition, detoxification, transformation, and excretion of xenobiotics in the organism (18, 19). The liver is critical in regulating lipid metabolism, amino acid metabolism, steroid metabolism, and many other metabolic pathways (20), of which metabolic disorders can result in severe damage and the promotion of function disturbances, for example, nonalcoholic fatty liver disease (NAFLD) (21). NAFLD covers the hepatic pathological change progression from steatosis to nonalcoholic steatohepatitis, fibrosis, and cirrhosis (20, 22–24), and can notably increase the risk of some more metabolic diseases such as diabetic cardiovascular disease and cancer (17, 24).

Previous research indicated that PM_2.5_ exposure affects various metabolic pathways *in vivo* (25), especially the pathways associated with lipid metabolism (18). Recent studies have shown that the liver has more obvious responses than the lung in metabolic disorders induced by PM_2.5_ exposure (26). PM_2.5_ exposure possibly contributes to dyslipidemia, vascular inflammation, lipid dysfunction, and insulin resistance, and then accelerates lipid-associated metabolic diseases such as atherosclerosis and type II diabetes mellitus (T2DM) (14, 27, 28). The impact of PM_2.5_ exposure on the liver has been getting more attention these years. Meanwhile, epidemiological studies suggested that airborne PM_2.5_ can improve the liver enzyme level (29), cause chronic liver inflammation, and increase the risk of cirrhosis and liver cancer (11, 30, 31). PM_2.5_ exposure has also been identified as an independent risk factor for NAFLD (21, 23). Previous toxicologic studies have shown that PM_2.5_ can transfer from the lung to the liver through multiple routes (26, 32), and it may expedite the progression of NAFLD through mechanisms such as inflammatory responses, oxidative stress, and insulin resistance (33, 34). PM_2.5_ exposure can induce insulin resistance (IR) *via* endothelial dysfunction, affecting hepatic insulin signaling pathways and suppressing the expression of peroxisome proliferator-activated receptors gamma (PPARγ) and PPARα, resulting in hepatic lipid accumulation (35, 36). Concurrently, PM_2.5_ exposure can cause oxidative stress, leading to hepatic tissue damage (37, 38). Moreover, exposure to PM_2.5_ may promote the expression of proinflammatory cytokines in adipocytesto and cause inflammation in NASH (39, 40).

This study randomly assigned C57BL/6J mice into two groups and treated them with PM_2.5_ suspension and PBS solution for eight weeks. The differences in liver tissue between the two groups, especially in metabolism and gene expression, were compared to investigate the impact of PM_2.5_ exposure on liver metabolism. The histopathological changes, differential expression of genes, and metabolites related to hepatic metabolic disorders were also analyzed to elaborate on the basic and comprehensive condition of the liver, illustrating the mechanism of injury triggered by PM_2.5_ exposure. To some extent, insights into hepatic metabolic disturbances benefit understanding the molecular pathology of metabolic diseases and exploring the potential therapeutic target.

## 2 Materials and methods

### 2.1 PM_2.5_ preparation and reagents

PM_2.5_ samples were collected and prepared according to previous studies (41), collected at Shanghai Jiao Tong University School of Medicine, Chongqing Road (S), Shanghai, China, from December 2017 to January 2018. The sampling site was close to the main traffic artery, and the surrounding area was densely populated. PM_2.5_ samples were collected on glass fiber filters for biological assay using a high-volume sampler (#2031, Lonying Company, Shandong, China) or quartz filters for chemical assay using a high-flow cascade sampler (#HFI 131, MSP Company, MN, USA) for 96 h. The collected PM_2.5_ sampling film was cut to the same size (3 cm × 3 cm) and randomly mixed. PM_2.5_ samples were split into four aliquots for chemical assay, which were analyzed for metal elements, polycyclic aromatic hydrocarbons (PAHs), elemental and organic carbon (EC and OC), and inorganic ion elements. The remaining sampling membranes were freeze-dried to separate PM_2.5_ for later animal experiments.

Metal elements in PM_2.5_ (Ca, Fe, Na, K, Al, Mg, Zn, Mn, Pb, Cu, Ba, Ti, Si, Sb, V, Mo, Sn, Ni, As, W, Cd) were measured by inductively coupled plasma mass spectrometry (# iCAP Q, ThermoFisher, MA, USA). Also, 16 PAHs (Naphthalene, Alenene, Acenaphthene, Indeno[1,2,3-cd]Pyrene, Benzo[*b*]Fluorathene, Fluorene, Pyrene, Benzo[*k*]Fluorathene, Benzo[*g,h,i*]Perylene, Fluoranthene, Benzo[*a*]Pyrene, Chrysene, Dibenz[*a,h*]Anthracene, Phenanthrene, Benz(*a*)Anthracene, Anthracene) were analyzed by gas chromatography-mass spectrometry (#*/7890A-5975C, Agilent, CA, USA). The inorganic ion elements (Ca^2+^, Na^+^, K^+^, Mg^2+^, SO_4_^2-^, NO_2_^-^, NO_3_^-^, Cl^-^, PO_4_^3-^) were analyzed by ion chromatography (#*ICS-5000+/900, Dionex, CA, USA). Thermophotometry detected the EC and OC content with a total organic carbon analyzer (#DRI Model 2015, DRI, NV, USA). The Instrumental Analysis Center of Shanghai Jiao Tong University provided the whole procedure of analysis and data. The results are shown in **Supplementary Table 1**.

PM_2.5_ samples were extracted for the biological assay by immersing the filters in ultrapure water and sonicating for 30 min (500 W, 40 kHz). Then they were recollected by a vacuum freeze drier (FreeZone2.5, Labconco Company, MO, USA). The extracted PM_2.5_ samples at 100 mg/mL concentration were stored at −80°C before animal and cell exposure.

### 2.2 Design of animal experiment

C57BL/6J mice (6 weeks old, n=12), purchased from the Animal Center of the Southern Model (Shanghai, China), were fed at relative temperature and humidity with a normal diet and water, providing ad libitum throughout the experiment. A total of twelve male mice were randomly divided into two groups, regardless of gender. After 2-week acclimation, mice in the PM_2.5_ group (n=6) were directly intranasally treated with 10 μL PM_2.5_ suspension (100 μg PM_2.5_/day) for 8 weeks, while the control group (n=6) were administered 10 μL phosphate buffer saline (PBS), which was in correspondence with the previous studies (38). When the exposure ended, all mice were subjected to a euthanasia procedure and then dissected. During the euthanasia procedure, we used the Rodent Anesthesia Machine (Gas Anesthesia, USA) with isoflurane as the anesthesia gas. We poured isoflurane into the anesthetic volatile tank, which was connected to the oxygen cylinder. Through the oxygen air blowing, volatile isoflurane was used with a concentration of 1.5-3%. The mice were anesthetized in 1-2 minutes and the body mass was measured. Then, liver samples were collected, weighed, and finally stored in a −80°C refrigerator for indicated experiments and analysis. Animal experiments have been approved by the animal and ethics review committee of the laboratory animal center at Shanghai Jiao Tong University School of Medicine (Shanghai, China).

### 2.3 Liver histological staining

Frozen liver tissues were cut into sections (3-5 μm) with the frozen slice system. Part of the​sections were stained with hematoxylin and eosin (H&E) for histology analysis according to standard protocols (#E607318, Sangon Biotech (Shanghai) Co., Ltd.). The tissue sections were stained with lipid droplets with freshly prepared Oil-Red-O solution (#E607319, Sangon Biotech (Shanghai) Co., Ltd.) and nuclei with hematoxylin (#E607318, Sangon Biotech (Shanghai) Co., Ltd.) to assess the lipid accumulation in liver tissues. The specimens were observed and photographed randomly in six fields of view by fluorescence microscopy (Olympus, Japan). Meanwhile, quantitative analysis of the Oil Red O positive area was analyzed with Image-Pro Plus 6.0 software (Media Cybernetics, Inc., USA), expressed as a percent.

### 2.4 Determination of hepatic TG and FFA levels

Hepatic TG and FFA levels were measured by the Triglyceride Detection Kit (#D799795, Sangon Biotech (Shanghai) Co., Ltd.), the Nonesterified Fatty Acid Detection Kit (#D799793, Sangon Biotech (Shanghai) Co., Ltd.) according to the manufacturer’s instructions, respectively. A BCA Protein Test kit (#C503021) was used to calibrate TG and FFA levels.

### 2.5 Quantitative real-time PCR analysis

Total RNA from frozen liver tissue samples was extracted by Trizol (Invitrogen, Carlsbad, CA, USA). The experiment was conducted in the same way as in our previous study (38). The target gene studied was *pparα*, while the reference gene was *β-actin*. The corresponding primer pairs are designed from Primerbank (https://pga.mgh.harvard.edu/primerbank/) as follows. *pparα*, forward primer: AGAGCCCCATCTGTCCTCTC; reverse primer: ACTGGTAGTCTGCAAAACCAAA; *β-actin*, forward primer: GGCTGTATTCCCCTCCATATATCG; reverse primer: CCAGTTGGTAACAATGCCATGT.

### 2.6 Protein level analysis

For immunofluorescence assay, frozen liver tissue sections were prepared and performed for 30 min at normal temperature before commencing with the staining protocol. Then, the slices were washed with PBS three times and blocked with 5% Bovine Serum Albumin (BSA) for 1 h. SREBP1 (#ab71983, 1:200, Abcam, USA) and PPARγ (#ab41928, 1:100, Abcam, USA) primary antibodies were incubated overnight. Slides were incubated with Anti-rabbit or anti-mouse IgG secondary antibody (#2975, #4408, 1:500, Cell Signaling Technology, USA) for 1 h, followed by nuclear staining with 4’,6-diamidino-2-phenylindole (DAPI) (#D9542, Sigma, USA) for 30 s. Mounted slides were observed using fluorescence microscopy (Olympus, Japan) to obtain the fluorescence images.

Western Blot analysis was operated as described in our previous studies (38). The primary antibodies included anti-SREBP1 (#ab71983, 1:1000, Abcam, USA), anti-PPARγ (#ab41928, 1:1000, Abcam, USA), and anti-PPARα (#ab41928, 1:1000, Abcam, USA). As loading control, β-Actin (#ab8226, 1:2000, Abcam, USA) was used.

### 2.7 Quantitative detection of acylcarnitine in liver

Acylcarnitine was profiled by liquid chromatography-tandem mass spectrometry, and the detailed method was described previously (42, 43). In brief, a total of 66 acylcarnitine were measured, including free carnitine (C0), 11 short-chain acylcarnitine (C2, C3, C3:0-OH, C4, C5, C5:0-2, C5:0-OH, C5:1, C6, C6-DC, C7:0-DC), 24 medium-chain acylcarnitine (C8, C8:0-OH, C10, C10-DC, C10:0-DC-OH, C10:1, C10:2-OH, C10:3-1, C10:3-DC, C12, C12:0-DC, C12:0-OH, C12-OH, C14, C14:0-DC, C14:0-OH, C14:1, C14:1-2, C14:1-DC-2, C14:2, C15:0-2, C15:2, C15:2-DC, and C15:2-OH), and 30 long-chain acylcarnitine (C16, C16:0-DC, C16:1, C16:1-DC, C16:1-OH, C16:2, C16:2-OH, C17, C17:1, C17:1-DC, C17:1-DC-OH, C17:2-OH, C17:3, C18, C18:0-OH, C18:1-OH, C18:2-OH, C20, C20:0-OH, C20:1, C20:1-OH, C20:2, C20:2-DC, C20:2-OH-1, C20:2-OH-2, C20:3, C20:3-OH, and C20:4).

### 2.8 High-resolution mass spectrometric analysis of liver sample

A total of eight mouse liver tissue samples were collected, and tissue preparation strictly followed the procedure described in the method METAB_NonTargeted_0001.00 (i.e., DMPA-labeling Kit for Amine & Phenol/Hydroxyl/Carboxyl Metabolomics I). The LC-MS analysis strictly followed the SOP (i.e., LC-MS Analysis for Dansyl-labeled Amine&Phenol/Hydroxyl/Carboxyl Metabolomics) using the HP-CIL Metabolomics Platform. Analysis was performed using IsoMS Pro 1.2.10 (NovaMT Inc.) and NovaMT Metabolite Database v1.0. A total of 8 samples assigned to 2 groups were uploaded to IsoMS Pro 1.2.10. Data Quality Checks and Data Processing were performed. Data were cleaned with peak pairs where Mean (sample)/Mean (blank) was less than or equal to 4.0 at a significance level of 0.05 and were filtered out. Peak pairs without data present in at least 50.0% of all samples and 80.0% of samples in any group were filtered out-the Ratio of Total Useful Signal normalized data. Metabolite identification was performed using the CIL library and LI library.

MetaboAnalyst 5.0 was used to analyze the differences between the control group and the PM_2.5_ exposure group in endogenous metabolites of the liver and to construct principal component analysis (PCA) and partial least squares-discriminant analysis (PLS-DA) models. Identifying the perturbed biological pathways on the differential metabolite data was performed using the Kyoto Encyclopedia of Genes and Genomes (https://www.kegg.jp, KEGG). Quantitative enrichment analysis (QEA) was performed using a generalized linear model to estimate a Q-statistic for each metabolite set, which describes the correlation between compound concentration profiles, X, and clinical outcomes, Y. All the pathways with adjusted *p*-value <0.05 were considered as the biological pathways perturbed by chronic exposure to PM_2.5_. Differential metabolites were defined according to the following criteria: FC > 1.5 or FC < 0.67 with a *p*-value < 0.05 and a q-value < 0.05.

### 2.9 High-throughput RNA sequencing

Total RNA was isolated and purified using TRIzol reagent (Invitrogen, Carlsbad, CA, USA) following the manufacturer’s procedure. Each sample’s RNA amount and purity were quantified using NanoDrop ND-1000 (NanoDrop, Wilmington, DE, USA). The RNA integrity was assessed by Bioanalyzer 2100 (Agilent, CA, USA) with RIN number >7.0 and confirmed by electrophoresis with denaturing agarose gel. Poly (A) RNA is purified from 1μg total RNA using Dynabeads Oligo (dT) 25-61005 (Thermo Fisher, CA, USA) using two rounds of purification. Then the poly (A) RNA was fragmented into small pieces using Magnesium RNA Fragmentation Module (NEB, cat.e6150, USA) for 94°C 5-7min. Then the cleaved RNA fragments were reverse-transcribed to create the cDNA by SuperScript™ II Reverse Transcriptase (Invitrogen, cat. 1896649, USA), which were next used to synthesize U-labeled second-stranded DNAs with E. coli DNA polymerase I (NEB, cat.m0209, USA), RNase H (NEB, cat.m0297, USA) and dUTP Solution (Thermo Fisher, cat.R0133, USA). An A-base is then added to the blunt ends of each strand, preparing them for ligation to the indexed adapters. Each adapter contains a T-base overhang for ligating the adapter to the A-tailed fragmented DNA. Single- or dual-index adapters are ligated to the fragments, and size selection was performed with AMPureXP beads. After the heat-labile UDG enzyme (NEB, cat.m0280, USA) treatment of the U-labeled second-stranded DNAs, the ligated products are amplified with PCR by the following conditions: initial denaturation at 95°C for 3 min; 8 cycles of denaturation at 98°C for 15s, annealing at 60°C for 15 sec, and extension at 72°C for 30 sec; and then final extension at 72°C for 5 min. The average insert size for the final cDNA library was 300 ± 50 bp. At last, we performed the 2×150 bp paired-end sequencing (PE150) on an Illumina Novaseq™ 6000 (LC-Biotechnology CO., Ltd., Hangzhou, China) following the vendor’s recommended protocol.

Raw data were filtered to remove the reads with the connector (adaptor), reads containing more than 5% ambiguous nucleotides, and low-quality reads (mean Q-value <20) using Cutadapt before analysis. Statistics of the raw sequencing amount, the effective sequencing amount, Q20, Q30, GC content, and comprehensive evaluation were performed. Total RNA was isolated and sequenced by Lc-Bio Technologies Co., Ltd. (Hangzhou, China). The data of RNA sequencing (RNA-SEQ) is stored in csv format after conversion, and the analysis of significant differences between samples and the relative quantification of the transcripts is performed by means of the DESeq2 package in R. The *p*-values of 0.05 were set as the threshold for significantly differential expression. The screening criteria for differentially expressed genes were defined as genes with FC > 1.50 or FC < 0.67 with *p*-value < 0.05. Meanwhile, pathway enrichment analysis was performed for differentially expressed genes by KEGG and Gene Ontology (GO). KEGG enrichment analyses of differentially expressed genes were implemented, and *p*-value<0.05 were considered significantly enriched.

### 2.10 Data processing and statistical analysis

The experimental data of acylcarnitine (ten samples in each group) were expressed as the Mean ± standard error of Mean (`Mean ± *SEM*) and analyzed with SPSS Statistics 26.0 software, using *Student-t* test (two-side). GraphPad Prism5 software was used for statistical analysis and bar plot across all staining counts data. Differences between the two groups were considered to be significant when the *p*-value < 0.05. The relationship between differential metabolites and differentially expressed genes. Cytoscape v3.9.0 was used to plot the network diagram for the selected parts with *a p*-value < 0.001. The correlation analysis between DEGs and DEMs was evaluated by the cor function in the R package and the network plot was performed with Cytoscape v3.9.0. The analysis of metabolomics and transcriptomics data, which was performed with four independent samples in each group (transcriptomics control group only with three groups) were generated with MetaboAnalyst 5.0 and OmicStudio (https://www.omicstudio.cn), showing with the PCA plots, volcano plots, advanced heatmap plots, and enrichment plots. The significant enriched biological pathways performed for DEMs and DEGs were screened with relative counts number, enrich factors and p-value of the selected enriched pathways. Information in detail about OmicStudio was shown elsewhere (44, 45).

## 3 Result

### 3.1 Chemical composition analysis of PM_2.5_

To evaluate the physical and chemical composition of PM_2.5_, metal elements, polycyclic aromatic hydrocarbons (PAHs), elemental carbon (EC) and organic carbon (OC), and inorganic ionic elements were detected. As illustrated in **Supplementary Table 1**, Calcium (9865 ng/mg), Sodium (3794 ng/mg), Iron (2418 ng/mg), and Potassium (1362 ng/mg) were the main elements in PM_2.5_. The content of Naphthalene was the highest and much higher than the other 14 PAHs, up to 6.73 ng/mg. Acenaphthene, Pyrene, and Benzo[*b*]Fluorathene were 0.89 ng/mg, 0.76 ng/mg, and 0.67 ng/mg, respectively. The cation and anion contents in PM_2.5_ were mainly as followings: NH_4_^+^ (3992 ng/mg), Ca^2+^ (1189 ng/mg), K^+^ (865 ng/mg), Mg^2+^ (147 ng/mg), SO_4_^2-^ (3767 ng/mg), NO_3_^-^ (2343 ng/mg), Cl^-^ (74 ng/mg), PO_4_^3-^ (68 ng/mg). The content of organic carbon (OC) and elemental carbon (EC) were 641 ng/mg and 177 ng/mg, respectively, as the ratio of OC/EC was 3.62.

### 3.2 Effects of PM_2.5_ exposure on histopathological analysis

**Figure 1:**
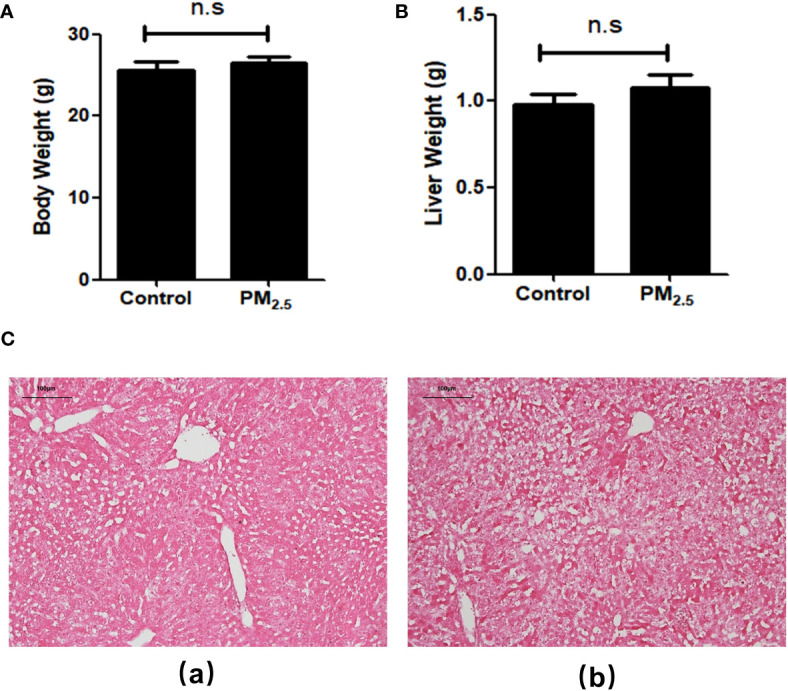
The Effect of PM_2.5_ exposure on the body weight, liver weight, and hepatic histology in mice. **(A)** Body weight; **(B)** Liver weight; **(C)** Representative pathological changes revealed by H&E staining of liver tissue. (a) Control; (b) PM_2.5_. Scale bar, 100 µm. n.s not significant.

After 8-week PM_2.5_ exposure, there was no significant difference in body weight (**Figure 1A**) and liver weight (**Figure 1B**) compared with the control group. To evaluate the effects of PM_2.5_ exposure on the liver of mice, we assessed the histological changes. As shown in the H&E staining results (**Figure 1C**), obvious pathological changes in the liver, including visible hepatic steatosis, irregular hepatic cords arrangement, and partial cytoplasmic vacuolation, can be seen in the PM_2.5_ exposure group, demonstrating the significant liver damage caused by PM_2.5_.

### 3.3 General analysis of hepatic metabolism profile after PM_2.5_ exposure

**Figure 2:**
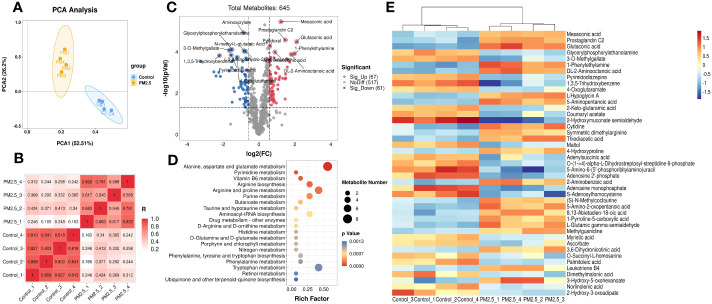
The Effect of PM_2.5_ exposure on the hepatic metabolites in mice. **(A)** PCA score plot. **(B)** Correlation analysis of all the biological samples. **(C)** Volcano plot of significant metabolites. **(D)** Cluster heatmap of differential metabolites. **(E)** KEGG enrichment analysis of differential metabolites. The y-axis represents the enrichment pathway, while the x-axis represents the enrichment ratio. The dot sizes represent the metabolites number. The color represents the p-value.

Then the metabolomics analysis of tissue was performed to investigate the effect of PM_2.5_ exposure on the liver. The principal components analysis (PCA) was carried out to visualize variation in identified metabolites between the PM_2.5_ exposure group and the control group (**Figure 2A**). The results showed that PC1 represents 52.51% of the variation, and 26.2% is represented by PC2. Correlation analysis was executed to test the relevance and repeatability between samples (**Figure 2B**). It can be seen that the PM_2.5_ exposure group was separated from the control group, indicating that endogenous substances in the PM_2.5_ exposure group have changed compared with the control group, manifested in some specific metabolites.

The total of 645 metabolites annotated by two groups are shown in the volcano plot (**Figure 2C**), which was constructed by plotting each metabolite’s fold change (FC) against the p-value. For comparison of the PM_2.5_ exposure group and the control group, the analysis showed 67 peak pairs with FC>1.5, *p*-value<0.05 (in red), and 61 peak pairs with FC<0.67, *p*-value< 0.05 (in blue). Hierarchical clustering analyses were used to elaborate on accumulating the 128 differential metabolites. **Figure 2E** shows the part of the top 43 differential metabolites between the two groups (FC>2 or FC<0.5, *p*-value< 0.05), and a more detailed view is shown in **Supplementary Figure 1**. Differential metabolites between the two groups are mainly amino acids and derivatives, lipids and lipid-like molecules, organic acids and derivatives, and nucleosides, nucleotides, and nucleotide analogs. The metabolites that decreased most significantly under PM_2.5_ exposure are 2-hydroxymuconate semialdehyde and 1,3,5-trihydroxy benzene. However, 1-Phenylethylamine, Glutaconic acid, Mesaconic acid, and Prostaglandin C2 increased remarkably (**Supplementary Figure 2**).

According to the KEGG analysis, there are a total of 37 KEGG pathways enriched in the PM_2.5_ exposure group (**Supplementary Table 2**). Moreover, the top 20 pathways include alanine, aspartate, glutamate metabolism, pyrimidine metabolism, vitamin B6 metabolism, arginine biosynthesis, arginine, proline, and purine metabolism etc, which are presented in **Figure 2D**. All these significantly enriched pathways mainly gather on amino acid and fatty metabolism, followed by glucose and vitamin metabolism. Six pathways (butanoate metabolism, fatty acid biosynthesis, arachidonic acid metabolism, biosynthesis of unsaturated fatty acids, linoleic acid metabolism, and α-Linolenic acid metabolism) are associated with fatty metabolism. Furthermore, amino sugar and nucleotide sugar metabolism, galactose metabolism, and citrate cycle (TCA cycle) were three pathways related to glucose metabolism.

### 3.4 General analysis of hepatic gene expression profiles after PM_2.5_ exposure

**Figure 3:**
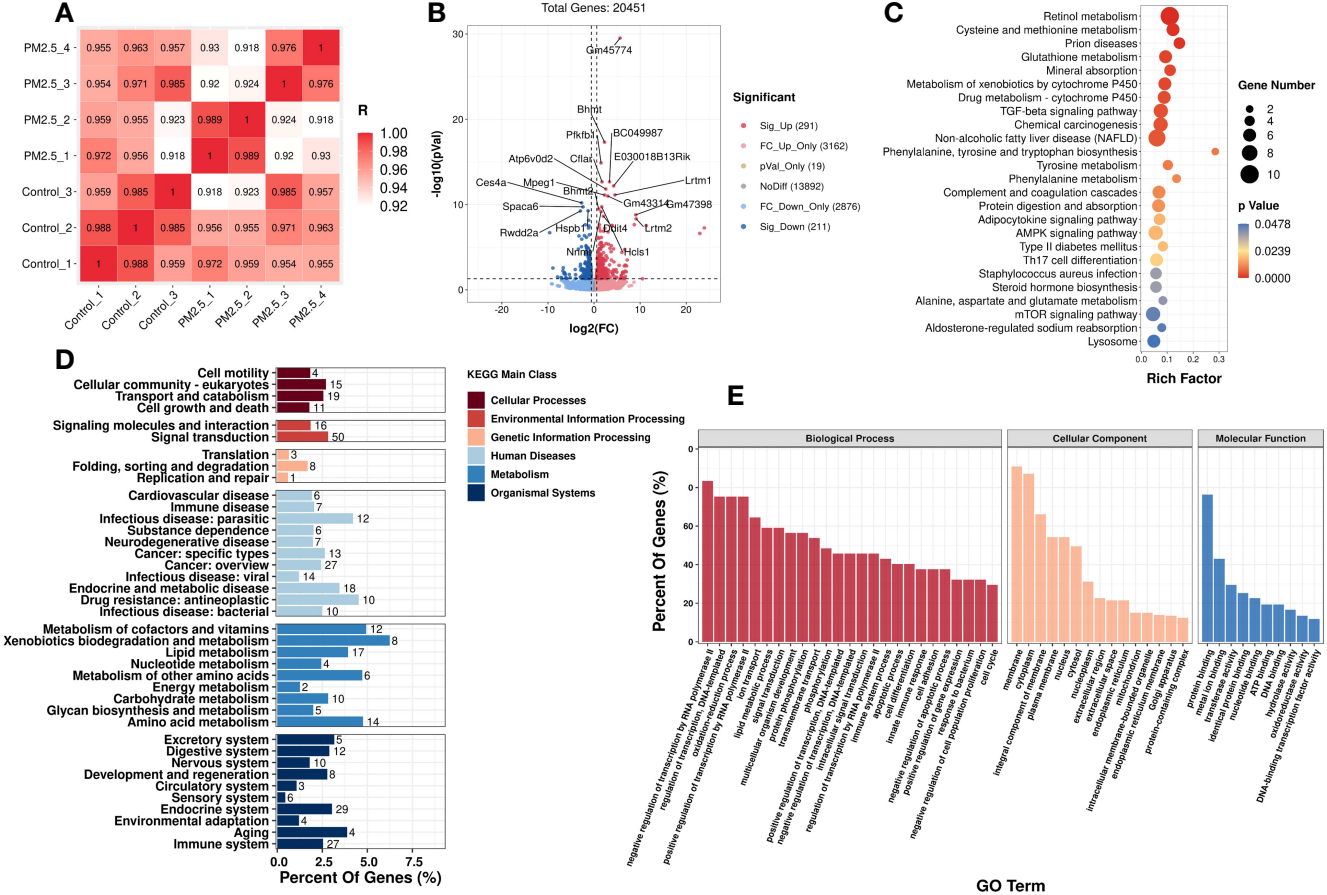
The effect of PM_2.5_ exposure on the hepatic gene expression in liver tissue. **(A)** Correlation analysis of all the biological samples. **(B)** Volcano plot showing the DEGs in the liver. KEGG **(D)** and GO **(E)** enrichment of class bar plot. The *y*-axis represents the enrichment of the subclass, while the *x*-axis represents a percent of genes. The bar’s color represents the different KEGG main classes (see color scale). **(C)** KEGG Enrichment scatter plot of DEGs. The *y*-axis represents the enrichment pathway, while the *x*-axis represents the rich factor. The dot sizes represent the gene number. The color represents the *p*-value.

In addition to the metabolomics analysis, transcriptomics analysis was performed to provide an overview of changes in hepatic gene profile after PM_2.5_ exposure and to explore the deeper effects of PM_2.5_ exposure on the liver. Correlation analysis was executed to test the relevance and repeatability between samples (**Figure 3A**). The results showed that the control samples were reproducible, while PM_2.5_ exposed samples had a high pairwise correlation.

Initially, setting the threshold as FC>1.5 or FC<0.67 with a *p*-value<0.05, a total of 502 differently expressed genes (DEGs) (291 upregulated DEGs and 211 downregulated DEGs) were found between the PM_2.5_ exposure group and control group (**Figure 3B**).

GO and KEGG analyses were performed to understand these DEGs’ functions better. It was found that 25 KEGG pathways (**Supplementary Table 3**) and 599 GO terms were significantly enriched, respectively. In **Figure 3E**, the top enriched GO terms are in three subclasses including biological process, cellular component, and molecular function. Meanwhile, 25 KEGG significantly enriched pathways are mainly related to fatty and amino acid metabolism. It is worth noting that PM_2.5_ exposure also significantly affected three signaling pathways: TGF-β signaling, AMPK signaling, and mTOR signaling. Moreover, the pathways of diseases related to metabolic disorders, such as NAFLD and T2DM, were also significantly enriched under PM_2.5_ exposure. The top 20 enriched pathways, according to the KEGG database, are shown in **Figure 3D**. We also performed a taxonomic analysis to demonstrate the proportion of significantly expressed genes in these DEGs subclasses. Under Metabolism terms, these DEGs mainly reacted in five main subclasses, including Metabolism of cofactors and vitamins, Xenobiotics biodegradation and metabolism, Amino acid metabolism, Metabolism of other amino acids, and lipid metabolism (**Figure 3C**).

### 3.5 Effects of PM_2.5_ exposure on hepatic levels of acylcarnitine

Combining the metabolomics and transcriptomics data, we found that, though several different pathways are involved, pathways related to lipid metabolism were significantly highlighted in both the gene expression and metabolites in the PM_2.5_ exposure group. Previous studies have proved that acylcarnitine, a kind of ester in carnitine, plays a key role in fatty acid oxidation. So, we measured the acylcarnitine levels in the liver tissue to observe the lipid metabolic level. The standardized quantitative detection result of acylcarnitine in the liver is presented in **Table 1**, presented in terms of mean ± standard error of the Mean, median, and minimum-maximum for each acylcarnitine profile of the PM_2.5_ exposure group and the control group. Sixty-six kinds of acylcarnitine were detected in the two groups, and 23 carnitine content significantly changed among them. Comparing acylcarnitine levels in control and PM_2.5_ exposure, groups revealed a significant decrease only in C14:1-2 (*p<0.05). Meanwhile, a significant increase was observed in average C0, C3:0-OH, C5:0-OH, C6-DC, C7:0-DC, C10:0-DC-OH, C10:2-OH, C10:3-DC, C14:0-DC, C15:2-DC, C16, C16:0-DC, C16:1, C16:1-DC, C17:1, C18, C18:1, C18:1-OH, C20:0, C20:0-OH, C20:2-OH-2, C20:3 levels (*p<0.05). There was no statistically significant difference in other acylcarnitine levels (*p*>0.05). The analysis of hepatic acylcarnitine levels showed that the lipid metabolism of the liver was disordered.

**Table 1:** Acylcarnitine values of liver tissue.

	Control	PM_2.5_	*p*
	Mean ± SEM	Mean ± SEM	
	Median(min-max)	Median(min-max)	
C0 (free carnitin)	4.93 ± 0.29	7.61 ± 0.61	0.002
	5.05(3.3-5.99)	8.5(4.23-9.32)	
C3:0-OH	0.24 ± 0.04	0.41 ± 0.04	0.01
	0.23(0.06-0.42)	0.46(0.2-0.57)	
C5:0-OH	0.34 ± 0.02	0.46 ± 0.04	0.022
	0.35(0.22-0.43)	0.41(0.34-0.74)	
C6-DC	1.34 ± 0.17	2.39 ± 0.26	0.003
	1.16(0.71-2.3)	2.45(1.3-4.02)	
C7:0-DC	0.41 ± 0.04	1.06 ± 0.1	<0.001
	0.38(0.28-0.63)	0.98(0.81-1.85)	
C10:0-DC-OH	0.02 ± 0	0.07 ± 0.01	<0.001
	0.02(0.01-0.03)	0.07(0.04-0.15)	
C10:2-OH	0.03 ± 0.01	0.06 ± 0.01	0.012
	0.03(0.01-0.07)	0.06(0.03-0.09)	
C10:3-DC	0.08 ± 0.01	0.22 ± 0.02	<0.001
	0.08(0.05-0.12)	0.21(0.15-0.39)	
C14:0-DC	0.06 ± 0.01	0.11 ± 0.01	<0.001
	0.07(0.03-0.1)	0.11(0.08-0.16)	
C14:1-2	0.1 ± 0.02	0.05 ± 0.01	0.037
	0.09(0.03-0.27)	0.04(0.03-0.08)	
C15:2-DC	0.05 ± 0.01	0.08 ± 0.01	0.015
	0.05(0.03-0.08)	0.08(0.04-0.13)	
C16	3.23 ± 0.44	6.71 ± 1.41	0.039
	2.99(1.5-5.74)	4.78(2.83-14.91)	
C16:0-DC	0.22 ± 0.02	0.38 ± 0.04	0.003
	0.21(0.13-0.31)	0.34(0.24-0.69)	
C16:1	0.92 ± 0.1	1.68 ± 0.23	0.009
	0.83(0.52-1.32)	1.47(0.79-2.92)	
C16:1-DC	0.05 ± 0.01	0.1 ± 0.01	<0.001
	0.05(0.03-0.08)	0.09(0.07-0.12)	
C17:1	0.04 ± 0	0.08 ± 0.01	0.015
	0.04(0.02-0.06)	0.06(0.04-0.14)	
C18	1.92 ± 0.26	4.16 ± 0.68	0.01
	1.87(0.86-3.78)	3.52(2.18-8.04)	
C18:1	5.54 ± 0.66	11.16 ± 2.08	0.026
	5.53(2.79-8.9)	7.64(5.4-22.47)	
C18:1-OH	1.35 ± 0.18	2.01 ± 0.22	0.032
	1.18(0.63-2.23)	1.86(1.11-3.29)	
C20:0	0.09 ± 0.01	0.19 ± 0.02	0.004
	0.08(0.04-0.19)	0.19(0.05-0.29)	
C20:0-OH	0.06 ± 0.01	0.12 ± 0.01	0.001
	0.06(0.04-0.09)	0.14(0.06-0.16)	
C20:2-OH-2	0.03 ± 0	0.05 ± 0.01	0.035
	0.03(0.02-0.05)	0.06(0.02-0.08)	
C20:3	0.45 ± 0.05	0.62 ± 0.06	0.044
	0.38(0.28-0.67)	0.53(0.36-1)	

SEM, Standard Error of Mean; min-max, minimum-maximum.

### 3.6 Correlation analysis on metabolomics and transcriptomics

**Figure 4:**
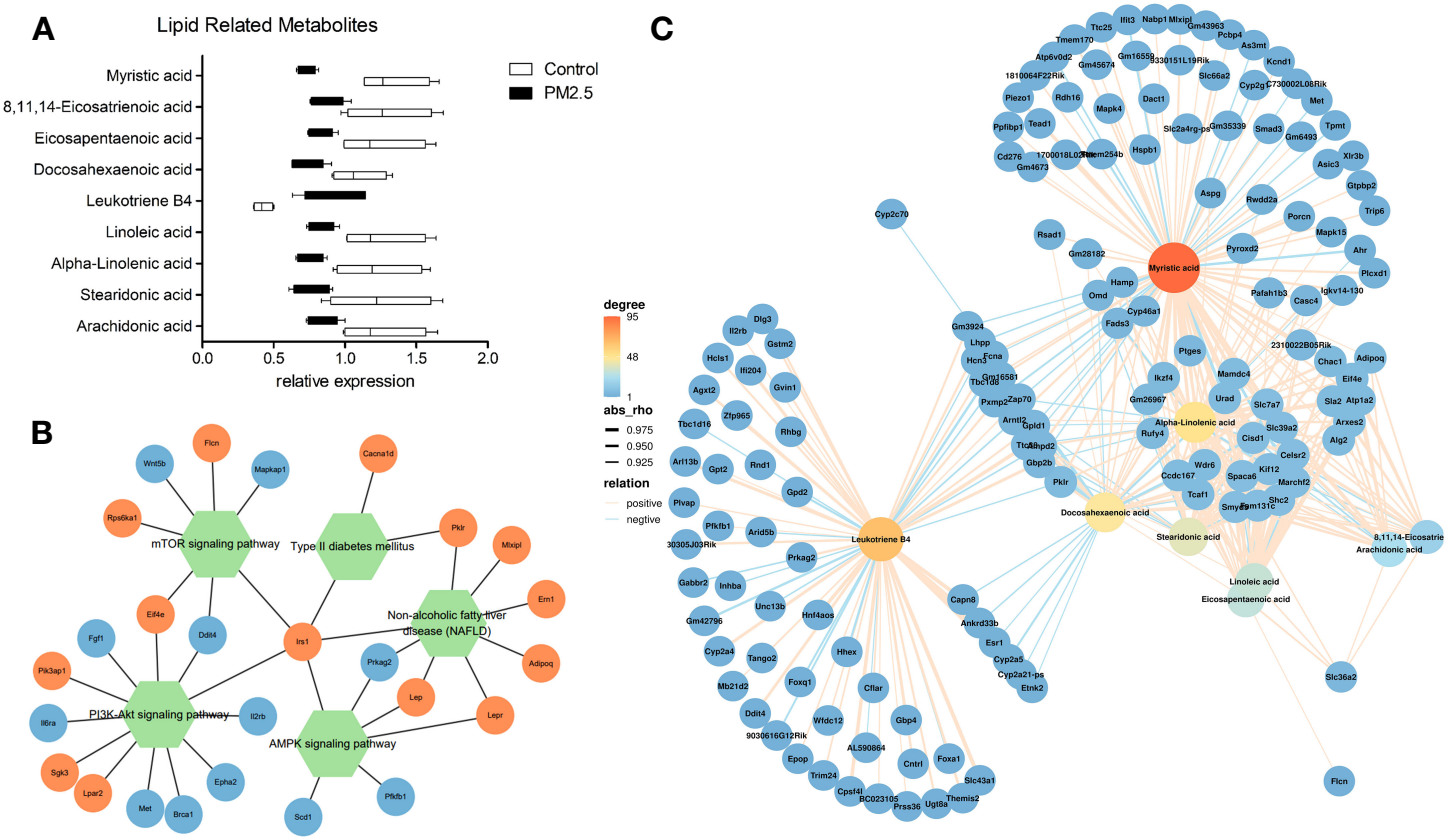
The effect of PM_2.5_ exposure on the correlation of DEMs and DEGs. **(A)** Relative expression of lipid metabolism-related DEMs. **(B)** Network(Pathway-Gene) of significant enrichment of DEGs by KEGG. **(C)** Association analysis of transcriptomic and metabolomic variation. Connection network between lipid metabolism-related DEMs and DEGs (rho > 0.80 and p-value < 0.005).

We further verified the relationship between metabolomics and transcriptomics under PM_2.5_ exposure. Significant lipid metabolism-related differential metabolites were detected through pathway enrichment analysis with differential metabolites. Besides Leukotriene B4, lipid metabolism-related metabolites were significantly reduced (**Figure 4A**). Moreover, two signaling pathways (mTOR/AMPK) and two disease-associated gene pathways (type 2 diabetes, NAFLD) were identified through the enrichment of DEGs by KEGG. We analyzed and observed the correlation of the enriched DEGs in these pathways and added one other top enriched signaling pathway (PI3K-Akt), which showed that the IRS1 gene played a central role and the pathways were interrelated and influenced each other (**Figure 4B**). By performing a correlation analysis between the global DEM and DEG, using the standard of rho > 0.5 and *p*-value < 0.05, the results showed a broad correlation between DEM and DEG (Data not shown). To further observe the changes and correlations related to lipid metabolism, the screened metabolites related to lipid metabolism were used as the main nodes. The divergent selection of related DEGs was performed using the analysis criteria of rho > 0.80 and *p*-value < 0.005. A significant clustering was observed, with two centers formed around Leukotriene B4 and other lipid metabolites (**Figure 4C**). We found that amino acid and carbohydrate metabolism in the KEGG pathway correlated (**Supplementary Figure 3**).

### 3.7 Effects of PM_2.5_ exposure on disorders of lipid metabolism

**Figure 5:**
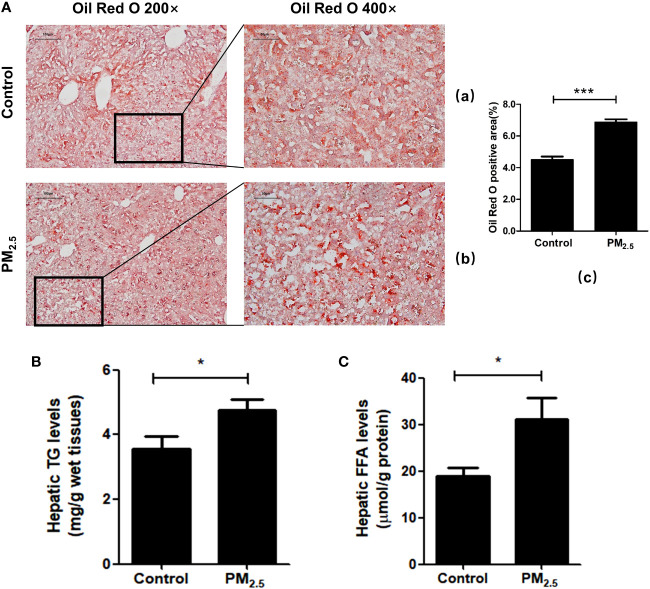
The effect of PM_2.5_ exposure on hepatic lipid accumulation in mice. **(A)** Liver pathology was assessed by Oil Red O staining. (a) Control; (b) PM_2.5_; (c) Quantification of the percentage of liver oil deposits. **(B)** Quantitative enzyme-based assays of hepatic triglycerides (TG) levels. **(C)** Quantitative enzyme-based assays of hepatic free fatty acids (FFA) levels. Data represent more than three independent experiments (Mean ± SEM). Scale bar, 100 µm, and 50 µm. n.s not significant. * *p* < 0.05, *** *p* < 0.001.

To further verify liver steatosis under PM_2.5_ exposure, we performed Oil Red O staining of mice liver tissue to measure concentrations of intracellular triglyceride stored in the lipid droplets of hepatic cells, which was contrasted with quantitative analysis of the ratio of oil red positive regions to total cell area (**Figure 5A**). The PM_2.5_-treated group had higher lipid accumulation in the hepatocytes than the control group (***p<0.001). In addition, increases in hepatic triglyceride (TGs) and FFA levels were also observed in PM_2.5_-exposed group (**Figures 5B, C**). These results suggest that ambient PM_2.5_ exposure results in the accumulation of lipids and elevation of FFA in the liver.

Furthermore, studies have shown that the AMPK signaling pathway, which is significantly enriched in the transcriptomics analysis of our data, may be one of the key pathways to regulating lipid metabolism and deposition. Related genes participate in adipogenesis, such as the activated sterol regulator-binding protein 1 (SREBP1) and the peroxisomal proliferative agent-activated receptor gamma (PPARγ). For further verification of lipid metabolic disorders, an immunofluorescence technique was performed to analyze the expression of SREBP1 and PPARγ.

**Figure 6:**
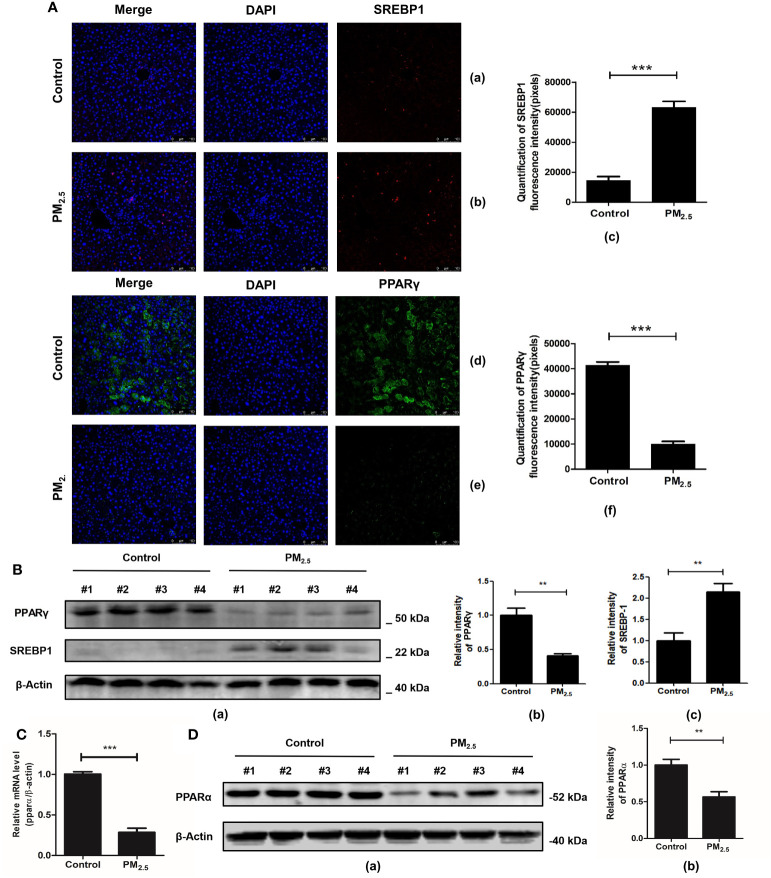
The effect of PM_2.5_ exposure on the expression of SREBP1, PPARγ, and PPARα in mice. **(A)** The immunofluorescence staining of SREBP1 and PPARγ. Nuclei were stained with DAPI. (a, d) Control; (b, e) PM_2.5_; (c, f) Graph quantifying SREBP1 and PPARγ. **(B)** The expression of PPARγ and SREBP1 after PM_2.5_ treatment. (a) Western blotting of PPARγ and SREBP1. (b, c) The quantification of the expression levels of PPARγ and SREBP1. **(C)** Quantitative real-time PCR analysis of PPARα mRNA expression. **(D)** The expression of PPARα protein in liver tissue. (a) Western blotting of PPARα. (b) The quantification of PPARα protein level. β-Actin as a reference gene. Data represent more than three independent experiments (Mean ± SEM). Scale bar, 100 µm. n.s not significant. ** *p* < 0.01, *** *p* < 0.001. n = 3 mice per group.

One of the key elements in hepatic metabolic disorder studies is SREBP1, a major lipogenic transcription factor. Over-activation and over-expression of SREBP1 can lead to an imbalance of lipid homeostasis, prone to triglyceride accumulation and cirrhosis (46). Consistent with previous research (47), significantly higher expressions of SREBP1 were revealed in the PM_2.5_ exposed group compared with the control group (*p*<0.001) (**Figure 6A**).

PPARγ is another key gene regulating fatty acid storage by activating genes that stimulate lipid ingestion and adipogenesis (48). Our study showed that mice in the PM_2.5_ exposure group decreased PPARγ expression significantly compared to the control group as detected by immunofluorescence assay (**Figure 6A**). To further verify the immunofluorescence results, western blot analysis was performed to detect the protein levels of SREBP-1 and PPAR-γ in hepatic tissues. The results showed that SREBP-1 was significantly upregulated, while the opposite results were observed in the PPARγ level (**Figure 6B**).

Considering that PPARα plays an important role in the regulation of liver lipid metabolism (49), the effect of PM_2.5_ exposure on PPARα expression was evaluated by qPCR and western blotting. As shown in **Figure 6C**, the level of PPARα mRNA in the liver of PM_2.5_ was significantly reduced (*p* < 0.01). Then, Western blotting was performed to confirm the downregulation of PPARα (**Figure 6D**). These results indicated that PM_2.5_-induced liver lipid metabolic disorders might be related to the downregulation of PPARα and PPARγ expression *via* deceleration of lipoprotein transport and increased lipotoxicity (50).

## 4 Discussion

Air pollution PM_2.5_ not only has a huge impact on the respiratory and cardiovascular systems but also seriously impacts the whole body and various organs through the plasma and into peripheral tissues (51). Many researches have confirmed the central function of the active oxygen (ROS) and inflammatory factors in the regulation of PM_2.5_ multi-organ toxicity. Because PM_2.5_ is abundant in active oxidants like metals, PAHs, and quinones (**Supplementary Table 1**), it can induce ROS to form through lung redox reactions by direct interaction between PM_2.5_ and pulmonary lining. Numerous experiments have also shown that PM_2.5_ is responsible for forming lipid peroxidation products, and inflammatory factors that diffuse from the lungs could be transferred through the plasma and into peripheral tissues, where they could cause oxidative stress, inflammatory response, and, thus, damage (3, 5, 26, 44, 52).

As the liver is the core organ for detoxifying exogenous chemicals, liver damage caused by PM_2.5_ exposure has attracted more and more attention in recent years (17, 25, 28, 29). PM_2.5_ could directly affect the process of normal hepatic function by enhancing inflammatory cytokines, ultimately elevating the risk of NAFLD by abnormal lipids metabolism (44, 45). Besides, PM_2.5_-caused destruction of tight junctions allows intestinal bacteria and their toxic derivatives to leak, leading to liver inflammation and even the development of NASH (53). A study by Wang (54) has illustrated that long-term PM_2.5_ exposure can contribute to enteral malnutrition and, subsequently, abnormal glucose metabolism, which leads to the transfer of lipopolysaccharides into the systemic circulation, exacerbating the process of NAFLD and T2DM (44). More detailed functions need further verification and exploration to provide new insight into our knowledge of the potential molecular mechanism of hepatic injury by PM_2.5_ exposure. Our study provides a comprehensive description and a partially targeted analysis of the differences in liver metabolism and gene expression under PM_2.5_ exposure, especially lipid metabolic disorders.

Previous studies have shown that dysregulation of hepatic lipid metabolism plays an important role in various metabolic diseases (24, 55). Nonalcoholic fatty liver disease, the most common liver pathological change, is characterized by lipid accumulation and is closely associated with metabolic syndrome (32, 33). Our study found that the pathways of NAFLD and T2DM were significantly enriched under PM_2.5_ exposure by the analyses of transcriptome data. In previous studies, PM_2.5_ exposure may accelerate the occurrence of lipid-related metabolic diseases (27), such as T2DM and NAFLD (21, 56), by inducing dyslipidemia (52, 57) and adipose dysfunction (4), while the results of our analysis provide correlative evidence of gene expression for it. The integrated metabolomics and transcriptomics results showed that amino acid, carbohydrate, and lipid metabolism were disordered (**Supplementary Figure 3**).

In this study, we further measured acylcarnitine levels in mice liver tissue, which is widely used to screen for metabolic diseases and identify some relevant biomarkers (19, 58). Acylcarnitine plays an important role in maintaining normal liver function. As a specific substrate for mitochondrial fatty acid β-oxidation, it can help liver cells transfer fatty acids into mitochondria to provide energy for the body (19, 59), which is one of the major pathways of lipid metabolism (60, 61). When mitochondrial function is damaged, acylcarnitine accumulates, which can be a practical indicator for predicting hepatotoxicity (62). Our results found a significant accumulation of short-chain, medium-chain, and long-chain acylcarnitine, consistent with a previous study finding that PM_2.5_ exposure resulted in a significant accumulation of medium-chain acylcarnitine (17). Acylcarnitine, with obvious differences and stability under PM_2.5_ exposure among medium-chain or long-chain acylcarnitine, can be further used as a biomarker to indicate liver damage. Only myristoyl carnitine (C14:1) was decreased in statistical significance, but there was no relevant research to show its special mechanism in hepatocytes. At the same time, acylcarnitine accumulation also represents mitochondrial disorders to some extent and plays a key role in nonalcoholic steatohepatitis (34).

PPARs, which act as nuclear hormone receptors, regulate the transcription of genes associated with lipid metabolism and glucose metabolism.It has three isoforms: PPARα, PPARβ/δ and PPARγ. Numerous researches have demonstrated that PPARα negatively affects the proinflammatory and Acute Phase Reaction (APR) signaling pathways, as observed in the Systemic Inflammatory, Atherogenic, and Nonalcoholic Fatty Liver Disease (NASH) models (63–67). As a key transcriptional regulator of adipogenesis, PPARγ plays a key role in lipid storage and lipid droplet formation (63, 68). For instance, previous studies have shown that exposure to PM_2.5_ inhibits PPARγ signal transduction (17), to which subsequent liver damage, such as triglyceride accumulation and hepatic steatosis (46), can be partly attributed (37). Zheng et al.’s studies showed that PM_2.5_ induced abnormal lipid balance and decreased PPARγ and PPARα expression *in vivo* (63) and *in vitro* (37). In the present study, we also found that PPARα and PPARγ were significantly inhibited under PM_2.5_ exposure, indicating an imbalance of lipid homeostasis induced by PM_2.5_. However, the related effect and mechanism of hepatic steatosis need to be further studied, which may become a key step to understanding the impact and prevention of PM_2.5_ on liver injury.

SREBP-1c is a major transcription factor that regulates hepatic *de novo* lipogenesis through insulin (69). Furthermore, SREBP1 molecules mainly provide the building block by inducing lipid synthesis in rapidly growing cells (46). Hepatic *de novo* lipogenesis can be reduced, and excessive lipid accumulation can be suppressed by modulating the AMPK/SREBP1c/FAS signaling pathway (70). The role of SREBP-1c was demonstrated in a transgenic mouse model overexpressing SREBP-1c in the liver, which leads to the development of hepatic steatosis due to increased lipogenesis (71). Our gene network (**Figure 4B**) shows that the core node IRS1 is also related to SREBP-1c. Previous studies have shown the decrease in IRS1 expression associated with increased fat synthesis and steatosis. Docking and phosphorylation of Irs1/Irs2 in the cell can activate downstream kinase cascades, such as the PI3K-Akt pathway. Akt, activated by the pathway, can further inhibit hepatic gluconeogenesis but simultaneously activate SREBP1c-mediated hepatic lipid metabolism (72). In our study, it was demonstrated by fluorescent staining that PM_2.5_ exposure leads to overexpression of SREBP1, promoting the development of steatosis, which is consistent with previous findings (47).

According to the KEGG database, we found that there were seven common significantly enriched pathways combining the metabolomics and transcriptomics analysis data between the PM_2.5_ exposure group and control group, including alanine, aspartate and glutamate metabolism, phenylalanine, tyrosine, and tryptophan biosynthesis, phenylalanine metabolism, retinol metabolism, tyrosine metabolism, cysteine, and methionine metabolism and glutathione metabolism. The thorough KEGG pathway of the relationship between differential metabolites and DEGs related to glutathione metabolism, retinol metabolism, alanine, aspartate and glutamate metabolism, and cysteine and methionine metabolism are shown in **Supplementary Figure 1**.

Glutamate is essential to adjust glutathione levels in the body as the important substrate for synthesizing glutathione, which can undergo intermediate conversion by glutamine (62). Our study significantly altered the expression of glutathione (GSH) and L-Glutamic acid at PM_2.5_ exposure. Although oxidized glutathione (GSSG) was not a significantly altered metabolite, the GSH/GSSG ratio decreased with PM_2.5_ exposure due to the significant decrease in GSH. The decreased ratio is the hallmark of oxidative stress in the hepatocytes (17, 73), which is consistent with the previous study that exposure to PM_2.5_ may cause oxidative stress in the liver (26, 74).

IRS1 is essential to regulate insulin-dependent glucose utilization and glycogen synthesis. The disruption of IRS1 signaling is a key and common mechanism in developing insulin resistance in population and laboratory studies (75–77). In our transcriptomic analysis, we found that the expression of the irs1 was significantly downregulated in the PM_2.5_-exposed group, suggesting that IRS1 might be an important pathway for preventing and intervening in PM_2.5_-accelerated metabolic liver diseases. This idea was supported by an earlier study, finding that total flavonoids alleviate PM_2.5_-induced NAFLD by modulating the IRS1/Akt and CYP2E1/JNK pathways (78). Leukotriene B4 (LB4) could activate LB4r1 in hepatocytes, leading to cellular insulin resistance (79). In a recent study, Li et al. suggest that the LB4/Lb4r1 axis promotes the development of NAFLD by enhancing lipogenesis in hepatocytes, potentially serving as a therapeutic target for NAFLD (80). In the metabolomic analysis, we found LB4 was significantly upregulated in the PM_2.5_-treated group, indicating that LB4/Lb4r1 axis might participate in the PM_2.5_-induced abnormal liver metabolism. Thus, IRS1 and LB4 might mediate the hepatic pathological impact of PM_2.5_ exposure, which provides promising directions for developing therapeutic interventions.

In addition, the other detrimental influence of PM_2.5_ exposure can also be discovered with non-negligible abnormal phenomena shown in our study, including inflammatory response, insulin resistance, apoptosis, fibrosis, cofactor-related and vitamin-related metabolic disorders (5, 37, 52, 66, 81). Since our experimental study used single-sex mice and the gender differences were not considered, which may be certain restrictions. In future studies, we will continue to strengthen research on female mice to analyze further the distinct effects of PM_2.5_ exposure in both genders. As the metabolism and gene expression change depicted in our study, the specific effects and mechanisms of those changes need further exploration and experimental verification. The results may provide a comprehensive foundation and new insight into our knowledge of lipid accumulation and chronic hepatic injury by PM_2.5_ exposure.

## 5 Conclusion

Our study demonstrated that PM_2.5_ could induce extensive metabolic disturbances, particularly significant in lipid and amino acid dysregulation, through *in vivo* experiments combined with metabolomics and transcriptomics analyses. Meanwhile, our results revealed lipid dysfunction and hepatic steatosis induced by PM_2.5_ exposure, manifested as acylcarnitine and lipid droplet accumulation. Furthermore, we speculated and detected several key transcription factors as the potential regulatory effects in lipid metabolic disorders, PPARα, PPARγ, and SREBP1, and found their aberrant expression in the PM_2.5_ exposure group. Our study provides a novel molecular and genetic basis for a better understanding of the mechanisms of hepatic metabolic disorders induced by PM_2.5_ exposure, which can provide new insights into the toxicology of liver lipid metabolic disorders associated with air pollution and the risk assessment of chronic liver diseases.

## Data availability statement

The original contributions presented in the study are publicly available. This data can be found here: https://github.com/Chenxiao-Zhang/RawData.

## Ethics statement

The animal study was approved by the animal and ethics review committee of the laboratory animal center at Shanghai Jiao Tong University School of Medicine (Shanghai, China). The study was conducted in accordance with the local legislation and institutional requirements.

## Author contributions

XD and CZ designed the study. CZ and TM drafted the manuscript. TM, JW, and DM conducted the PM2.5 collection and animal experiment. TM and CZ conducted liver histological staining. ML and XW completed the Quantitative Detection of Acylcarnitine. CZ, JW, and CL processed high-resolution mass spectrometric analysis and high-throughput RNA sequencing. CZ, JR, and XW contributed to virtualization. CZ, CL, DM, JW, and ML contributed to data analysis and manuscript writing. All authors contributed to the article and approved the submitted version.
