# Supplementary material for: PM2.5 induced liver lipid metabolic disorders in C57BL/6J mice
Source: Front Endocrinol (Lausanne). 2023 Sep 14;14:1212291. doi: 10.3389/fendo.2023.1212291 (PMC10539470; doi:10.3389/fendo.2023.1212291)
Supplement: Supplementary file 1 [file DataSheet_1.doc]

Supplementary Information

**PM_2.5_ Induced Liver Lipid Metabolic Disorders in C57BL/6J mice**

Chenxiao Zhang^1, #^, Tengfei Ma^2, #^, Chang Liu^1^, Ding Ma^2^, Jian Wang^2, 3^, Meng Liu^2^, Jinjun Ran^1^,*, Xueting Wang^4^,*, Xiaobei Deng^1^,*

^1^ School of Public Health, Shanghai Jiao Tong University School of Medicine, Shanghai, China; ^2^ College of Basic Sciences, Shanghai Jiao Tong University School of Medicine, Shanghai, China; ^3^ Department of Cardiology, Renji Hospital, Shanghai Jiao Tong University School of Medicine, Shanghai, 200127, China; ^4^ Department of Cardiology, Tongren Hospital, Shanghai Jiao Tong University School of Medicine, Shanghai, China

**Supplementary Table 1.**

**Chemical characteristics of PM_2.5_**

| Metallic Elements | Concentration (ng/mg) | PAHs | Concentration (ng/mg) |
| --- | --- | --- | --- |
| Ca | 9863.586 | Naphthalene | 6.733 |
| Na | 3793.517 | Acenaphthene | 0.888 |
| Fe | 2418.193 | Pyrene | 0.761 |
| Mg | 1422.193 | Benzo[*b*]Fluorathene | 0.674 |
| K | 1362.290 | Acenaphthene | 0.547 |
| Si | 1254.897 | Indeno[1,2,3-cd]Pyrene | 0.503 |
| AI | 1149.876 | Fluoranthene | 0.405 |
| Zn | 379.297 | Fluorene | 0.405 |
| Mn | 131.228 | Chrysene | 0.341 |
| Ba | 94.138 | Benzo[*k*]Fluorathene | 0.285 |
| Cu | 92.083 | Benzo[*g,h,i*]Perylene | 0.278 |
| Ti | 59.600 | Philippines | 0.196 |
| Pb | 59.531 | Benzo[*a*]Pyrene | 0.179 |
| V | 40.952 | Dibenz[*a,h*]Anthracene | 0.149 |
| Sr | 38.386 | Benz(*a*)Anthracene | 0.105 |
| Mo | 16.745 |  |  |
| Ni | 16.234 | Anions | Concentration (ng/mg) |
| Sn | 11.793 | SO_4_^2-^ | 3766.992 |
| As | 10.676 | NO^3-^ | 2343.459 |
| B | 6.262 | Cl^-^ | 74.024 |
| Sb | 5.172 | PO_4_^3-^ | 67.784 |
| Zr | 4.717 | Cations | Concentration (ng/mg) |
| W | 4.372 | NH_4_^+^ | 3992.080 |
| Se | 3.476 | Ca^2+^ | 1189.000 |
| Bi | 3.310 | K^+^ | 864.880 |
| Li | 3.048 | Mg^2+^ | 146.960 |
| Hg | 2.524 |  |  |
| Co | 1.600 | Carbonaceous Components | Concentration (ng/mg) |
| Cd | 1.572 | Organic Carbon | 640.944 |
| Be | 0.110 | Elemental Carbon | 177.048 |

**Supplementary Table 2**

**Significant enrichment of KEGG pathways of differential metabolites**

| Pathway Name | Hits | Total Compound | Statistic Q | Raw  p value | FDR |
| --- | --- | --- | --- | --- | --- |
| Alanine, aspartate and glutamate metabolism | 8 | 28 | 83.708 | <0.001 | <0.001 |
| Pyrimidine metabolism | 1 | 39 | 93.214 | <0.001 | 0.002 |
| Vitamin B6 metabolism | 2 | 9 | 87.562 | <0.001 | 0.002 |
| Arginine biosynthesis | 4 | 14 | 85.983 | <0.001 | 0.002 |
| Arginine and proline metabolism | 6 | 38 | 84.676 | <0.001 | 0.002 |
| Purine metabolism | 4 | 65 | 80.485 | <0.001 | 0.002 |
| Butanoate metabolism | 2 | 15 | 85.031 | 0.001 | 0.002 |
| Taurine and hypotaurine metabolism | 1 | 8 | 88.252 | 0.001 | 0.002 |
| Aminoacyl-tRNA biosynthesis | 4 | 48 | 82.959 | 0.001 | 0.002 |
| Drug metabolism - other enzymes | 1 | 39 | 87.277 | 0.001 | 0.002 |
| D-Arginine and D-ornithine metabolism | 1 | 4 | 87.03 | 0.001 | 0.002 |
| Histidine metabolism | 1 | 16 | 85.856 | 0.001 | 0.002 |
| D-Glutamine and D-glutamate metabolism | 2 | 6 | 85.856 | 0.001 | 0.002 |
| Porphyrin and chlorophyll metabolism | 1 | 30 | 85.856 | 0.001 | 0.002 |
| Nitrogen metabolism | 1 | 6 | 85.856 | 0.001 | 0.002 |
| Phenylalanine, tyrosine and tryptophan biosynthesis | 2 | 4 | 83.967 | 0.001 | 0.002 |
| Phenylalanine metabolism | 2 | 10 | 77.737 | 0.001 | 0.002 |
| Tryptophan metabolism | 6 | 41 | 77.114 | 0.001 | 0.002 |
| Retinol metabolism | 1 | 17 | 84.353 | 0.001 | 0.002 |
| Ubiquinone and other terpenoid-quinone biosynthesis | 1 | 9 | 84.091 | 0.001 | 0.002 |
| Tyrosine metabolism | 1 | 42 | 84.091 | 0.001 | 0.002 |
| Cysteine and methionine metabolism | 3 | 33 | 81.513 | 0.001 | 0.002 |
| Glutathione metabolism | 3 | 28 | 71.552 | 0.002 | 0.003 |
| Glyoxylate and dicarboxylate metabolism | 2 | 32 | 68.978 | 0.003 | 0.004 |
| Fatty acid biosynthesis | 1 | 47 | 77.265 | 0.004 | 0.006 |
| beta-Alanine metabolism | 1 | 21 | 76.326 | 0.005 | 0.006 |
| Valine, leucine and isoleucine degradation | 2 | 40 | 72.713 | 0.005 | 0.006 |
| Arachidonic acid metabolism | 2 | 36 | 63.321 | 0.007 | 0.009 |
| Galactose metabolism | 1 | 27 | 67.693 | 0.012 | 0.015 |
| Glycine, serine and threonine metabolism | 1 | 33 | 66.982 | 0.013 | 0.016 |
| Amino sugar and nucleotide sugar metabolism | 1 | 37 | 64.856 | 0.016 | 0.018 |
| Citrate cycle (TCA cycle) | 1 | 20 | 63.912 | 0.017 | 0.019 |
| Folate biosynthesis | 1 | 27 | 58.931 | 0.026 | 0.029 |
| Biosynthesis of unsaturated fatty acids | 6 | 36 | 55.641 | 0.032 | 0.034 |
| Linoleic acid metabolism | 1 | 5 | 54.926 | 0.035 | 0.036 |
| alpha-Linolenic acid metabolism | 2 | 13 | 54.37 | 0.036 | 0.036 |

**Supplementary Table 3**

**Significant enrichment of KEGG pathways of DEG_S_**

| Pathway Name | Significant Gene Number | Total Gene Number | p value |
| --- | --- | --- | --- |
| Retinol metabolism | 10 | 91 | 0.00 |
| Cysteine and methionine metabolism | 6 | 49 | 0.00 |
| Prion diseases | 5 | 34 | 0.00 |
| Glutathione metabolism | 6 | 64 | 0.00 |
| Mineral absorption | 5 | 45 | 0.00 |
| Metabolism of xenobiotics by cytochrome P450 | 6 | 66 | 0.00 |
| Drug metabolism - cytochrome P450 | 6 | 68 | 0.00 |
| TGF-beta signaling pathway | 7 | 93 | 0.00 |
| Chemical carcinogenesis | 7 | 94 | 0.00 |
| Non-alcoholic fatty liver disease (NAFLD) | 9 | 149 | 0.00 |
| Phenylalanine, tyrosine and tryptophan biosynthesis | 2 | 7 | 0.01 |
| Tyrosine metabolism | 4 | 39 | 0.01 |
| Phenylalanine metabolism | 3 | 22 | 0.01 |
| Complement and coagulation cascades | 6 | 88 | 0.01 |
| Protein digestion and absorption | 6 | 90 | 0.01 |
| Adipocytokine signaling pathway | 5 | 71 | 0.02 |
| AMPK signaling pathway | 7 | 126 | 0.02 |
| Type II diabetes mellitus | 4 | 48 | 0.02 |
| Th17 cell differentiation | 6 | 102 | 0.02 |
| Steroid hormone biosynthesis | 5 | 88 | 0.04 |
| Staphylococcus aureus infection | 5 | 88 | 0.04 |
| Alanine, aspartate and glutamate metabolism | 3 | 36 | 0.04 |
| mTOR signaling pathway | 7 | 154 | 0.05 |
| Aldosterone-regulated sodium reabsorption | 3 | 38 | 0.05 |
| Lysosome | 6 | 124 | 0.05 |

**Supplementary Figure 1**


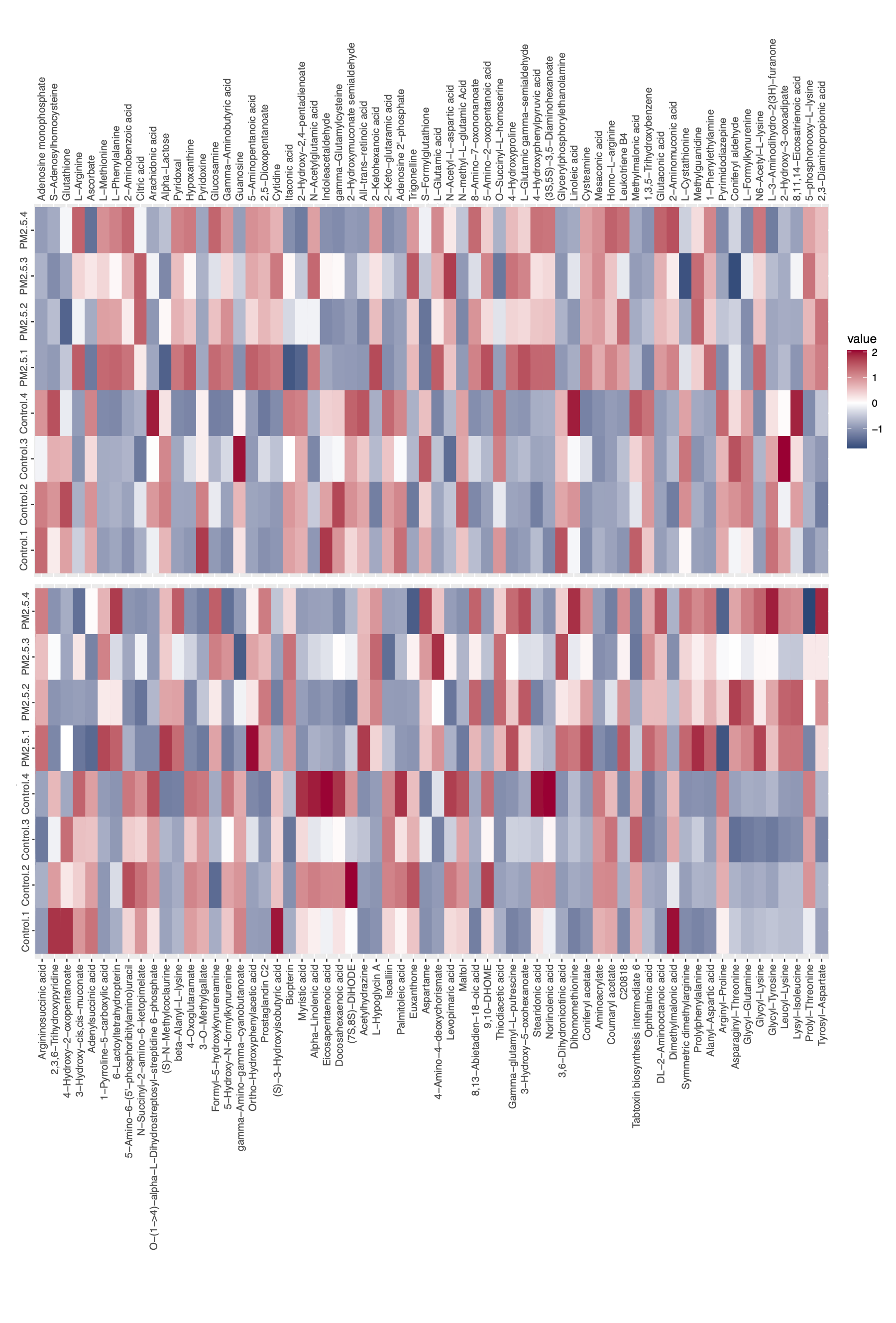


Cluster heatmap of 128 differential metabolites between the PM_2.5_ exposure group and the control group. (FC>1.50 or FC<0.66, p-value< 0.05)

**
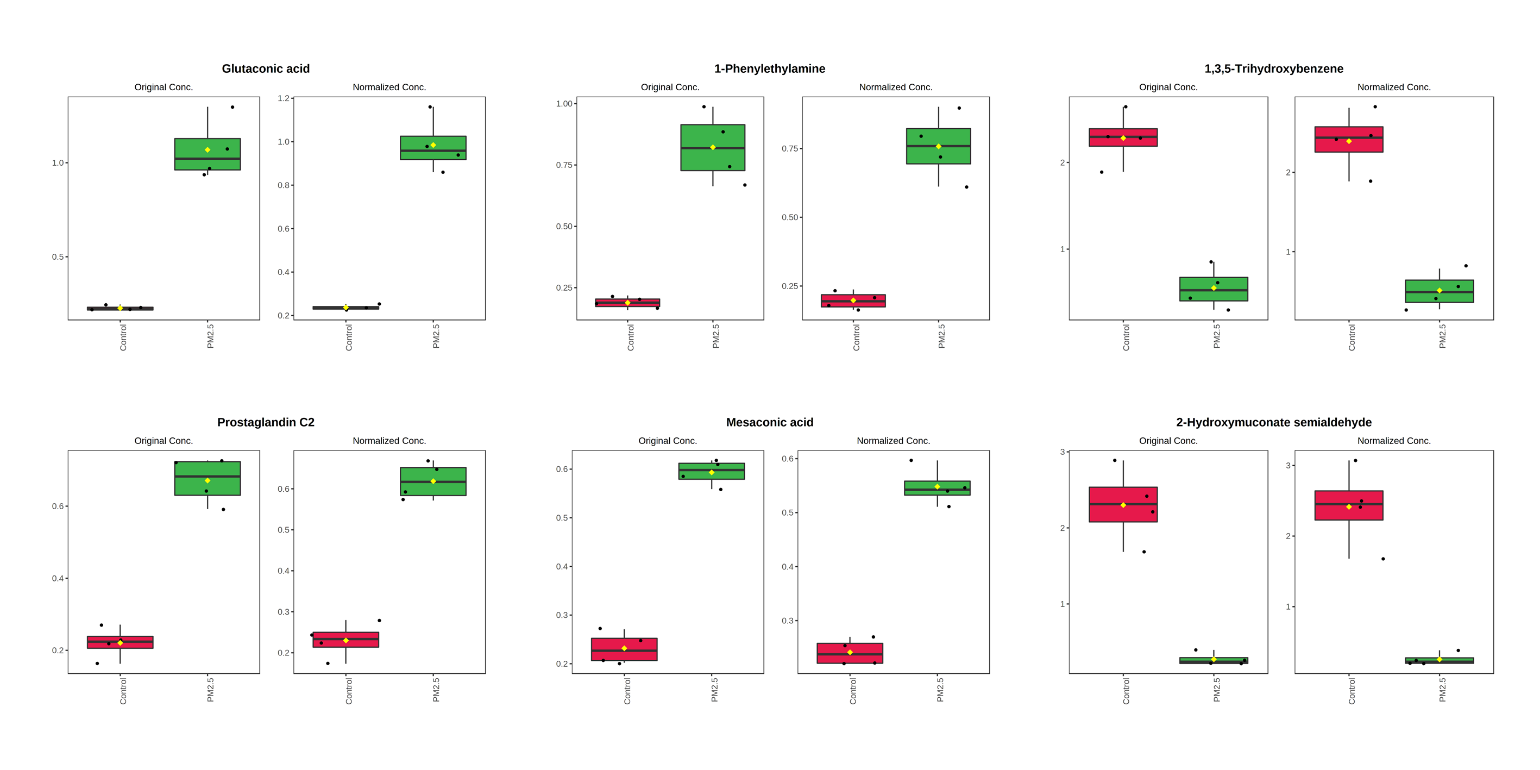
Supplementary Figure 2**

Significant differential metabolites select from volcano plot between the PM_2.5_ exposure group and the control group.


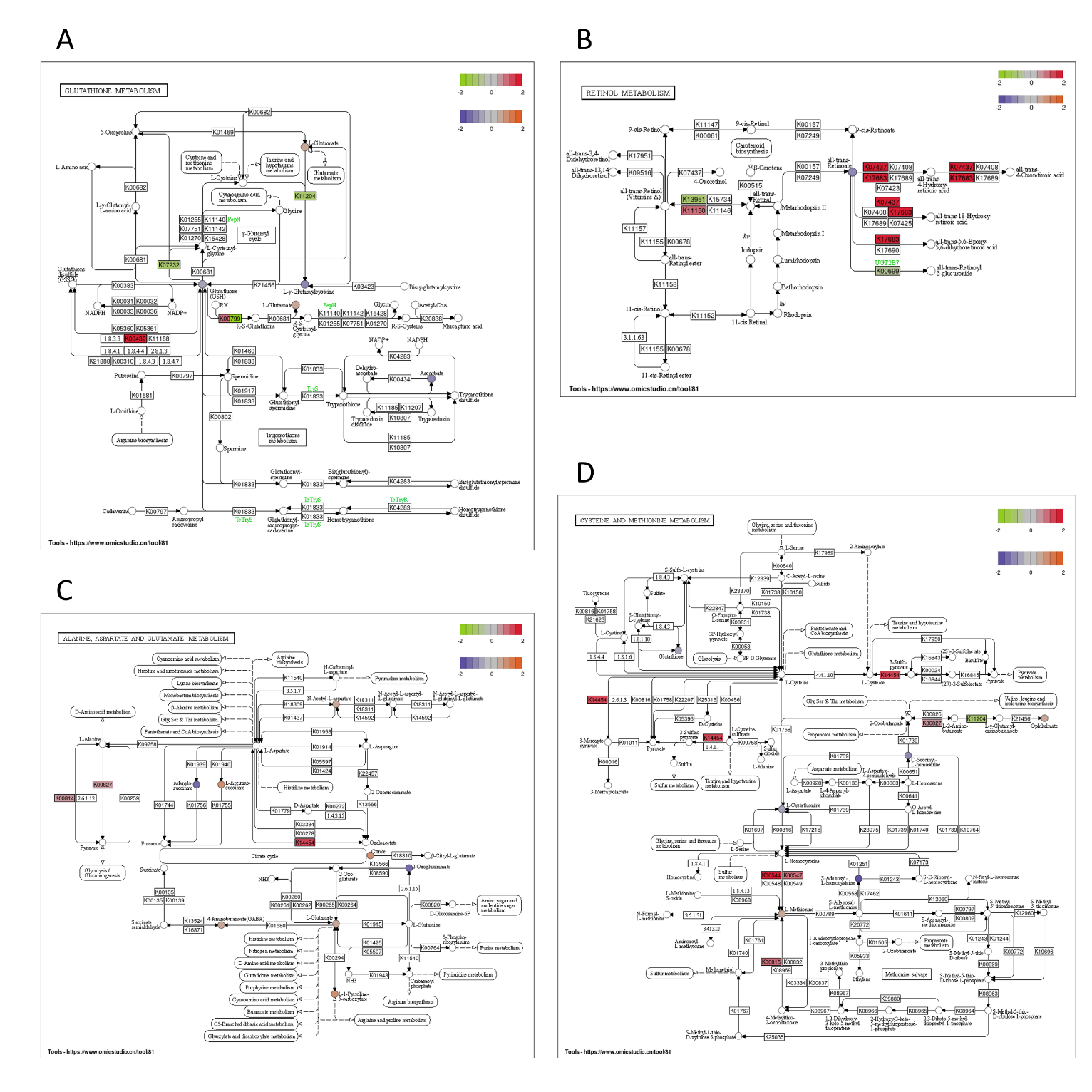
**Supplementary Figure 3**

The connected description of the DEMs and DEGs in part of the significant KEGG pathway.
